# Estimating the optimal perioperative chemotherapy utilization rate for muscle‐invasive bladder cancer

**DOI:** 10.1002/cam4.2449

**Published:** 2019-08-31

**Authors:** Safiya Karim, William J. Mackillop, Kelly Brennan, Yingwei Peng, D. Robert Siemens, Monika K. Krzyzanowska, Christopher M. Booth

**Affiliations:** ^1^ Department of Oncology Tom Baker Cancer Centre University of Calgary Calgary Canada; ^2^ Department of Oncology Queen's University Kingston Canada; ^3^ Department of Public Health Sciences Queen's University Kingston Canada; ^4^ Division of Cancer Care and Epidemiology Queen's University Cancer Research Institute Kingston Canada; ^5^ Department of Urology Queen's University Kingston Canada; ^6^ Division of Medical Oncology & Hematology Princess Margaret Cancer Centre Toronto Canada

**Keywords:** benchmarking, bladder cancer, neoadjuvant chemotherapy

## Abstract

**Background:**

Identifying optimal chemotherapy (CT) utilization rates can drive improvements in quality of care. We report a benchmarking approach to estimate the optimal rate of perioperative CT for muscle‐invasive bladder cancer (MIBC).

**Methods:**

The Ontario Cancer Registry and linked treated records were used to identify neoadjuvant and adjuvant CT rates among patients with MIBC during 2004‐2013. Monte Carlo simulation was used to estimate the proportion of observed rate variation that could be due to chance alone. The criterion‐based benchmarking approach was used to explore whether social and health‐system factors were associated with CT rates. We also used the “pared‐mean” approach to identify a benchmark population of hospitals with the highest treatment rates. Hospital CT rates were adjusted for case mix and simulated using a multi‐level multivariable model and a parametric bootstrapping approach.

**Results:**

The study population included 2581 patients; perioperative CT was delivered to 31% (798/2581). Multivariate analysis showed that treatment was strongly associated with patient socioeconomic status and hospital teaching status. The benchmark rate was 36%. Unadjusted CT rates were significantly different across hospitals (range 0%‐52%, *P* < .001). The unadjusted benchmark perioperative CT rate was 45% (95% CI 40%‐50%); utilization rate in nonbenchmark hospitals was 28% (95% CI 26%‐30%). When using simulated CT rates adjusted for case mix, the benchmark CT rate was 41% (95% CI 35%‐47%) and the nonbenchmark hospital CT rate was 30% (95% CI 28%‐32%). The simulation analysis suggested that the observed component of variation (38%) was outside the 95% CI (22%‐28%) of what could be expected due to chance alone.

**Conclusions:**

There is significant systematic variation in perioperative CT rates for MIBC across hospitals in routine practice. The benchmark perioperative CT rate for MIBC is 36%‐41%.

## Graphical abstract

Identifying optimal chemotherapy (CT) utilization rates can drive improvements in quality of care. In this benchmarking study of neoadjuvant chemotherapy for muscle‐invasive bladder cancer (MIBC), we found that treatment was strongly associated with patient socioeconomic status and hospital teaching status. The benchmark rate of neoadjuvant chemotherapy for MIBC was 36%‐41%.

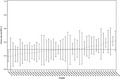

## 1 INTRODUCTION

More than half of patients with muscle‐invasive bladder cancer (MIBC) who undergo radical cystectomy will recur and die from their disease.^1^ A number of randomized controlled trials and meta‐analyses have demonstrated that neoadjuvant chemotherapy (NACT) is associated with a 5%‐10% absolute improvement in overall survival.^2^, ^3^, ^4^ Adjuvant chemotherapy (ACT) may also improve survival.^5^, ^6^ Despite recommendations by international guidelines, a number of studies have shown that rates of perioperative chemotherapy (CT) remain low in routine practice.^7^, ^8^ Our group has previously reported that rates of NACT and ACT in Ontario are 4% and 18%, respectively.^9^ More recent studies have shown that the utilization is increasing.^10^, ^11^ During 2009‐2013, utilization rates of NACT and ACT in Ontario were 19% and 20% respectively.^12^ Interpretation of this data is limited by the fact that the optimal utilization rate is not known. Without knowing the optimal perioperative CT utilization rate it is not possible to identify shortfalls in utilization and therefore not possible to close the gap between evidence and practice.^13^

Two methods are used to determine benchmark performance. Criterion‐based benchmarking (CBB) is an empirical method for estimating the appropriate rate of the use of a specific therapy that does not require comprehensive information about case mix at the population level.^14^ This approach has been used to estimate the proportion of patients who need radiotherapy.^15^, ^16^, ^17^, ^18^ The University of Alabama at Birmingham's Achievable Benchmarks of Care (ABC^TM^s) derives a “pared‐mean,” defined as the mean of the best care achieved for at least 10% of the population.^19^ This methodology is widely used in quality improvement initiatives. The objective of our study was to use the CBB and ABC methods to estimate a benchmark rate for the utilization of perioperative chemotherapy for MIBC.

## 2 METHODS

### 2.1 Study population

The study population included all patients who underwent cystectomy MIBC in Ontario between 2004 and 2013. Ontario has a population of approximately 13.5 million and a single‐payer universal health care system.

### 2.2 Data sources and linkages

The Ontario Cancer Registry (OCR) is a population‐based cancer registry that captures ~98% of all incident cases of cancer in Ontario.^20^, ^21^ Surgical interventions were identified from the Canadian Institute for Health Information. Physician billing records from the Ontario Health Insurance Plan and electronic treatment records were used to identify chemotherapy utilization. Surgical pathology reports were obtained from the OCR and reviewed by a team of trained abstractors.

### 2.3 Definition of variables

Teaching hospitals were affiliated with a medical school and routinely had residents on service. Hospitals that delivered chemotherapy were classified as having medical oncology on‐site. Ontario's regional cancer centres (RCC) are comprehensive cancer centres with on‐site radiation facilities; and during the study period there were 13 RCCs. Indicators of the socioeconomic status (SES) of the community in which patients resided at diagnosis were linked as described previously.^22^ Rurality was determined using postal code and based on living in a municipality with fewer than 10 000 people. Comorbidity was classified using the modified Charlson Index.^23^

### 2.4 Outcome measures

Perioperative chemotherapy was defined as patients receiving CT within 16 weeks prior (neoadjuvant) and/or 16 weeks after cystectomy (adjuvant). The degree of variation of perioperative CT was described by the coefficient of variation (CV) of the hospital‐specific rates. When actual rates are lower than the benchmark rate, the unmet need for perioperative CT is measured in terms of the “shortfall,” where: %shortfall = (benchmark rate − actual rate)/benchmark rate × 100%. Adjusted survival rates were estimated using a Cox regression model.

### 2.5 Quantifying the random component of variation in perioperative CT rates

Monte Carlo simulation was used to determine the degree of interhospital variation in perioperative CT rates that would be expected due to chance alone, as described previously.^24^ The simulation model assumed that the probability of use of perioperative CT was the same at every hospital, and equal to the observed probability of use of perioperative CT in the overall study population. The model used the actual number of hospitals in Ontario and the actual number of patients seen at each hospital. We did 1000 iterations of the model. The CV of hospital‐specific perioperative CT rates was calculated for each set of simulated data. The mean and 95% CI of the 1000 simulated CVs was used to quantify the degree of variation in hospital‐specific perioperative CT rates that would be expected due to chance alone in this study population. The magnitude of the nonrandom component of variation in perioperative CT rates was estimated by subtracting the expected CV from the observed CV.

### 2.6 CBB: Criterion‐based benchmarking method

The CBB process used had four steps: (a) logistic regression to identify social and health system‐related factors that impede access to perioperative CT; (b) identification of a benchmark subpopulation with unimpeded access to perioperative CT; (c) measurement of the perioperative CT rate in the benchmark population; and (d) direct standardization of the benchmark rate to the case mix of the general cancer population.^25^

### 2.7 ABC method

The ABC method is operationalized using the “pared‐mean” approach. To create benchmark levels, hospitals were ranked on descending order of rates of perioperative CT. We then removed those hospitals with <10 cases. Beginning with the best performing hospital, patients in each hospital were then summed until the combined population of this subset was at least 10% of the entire study population. Pooling patients from these top‐performing hospitals, the benchmark rate was then calculated as the proportion of patients who received perioperative CT in this subset of hospitals.^19^

To adjust perioperative CT rates for case mix, a multi‐level multivariable logistic regression model accounting for known patient‐ and disease‐related characteristics associated with perioperative CT and random variation at the level of hospital was employed. The predicted probability of each patient receiving perioperative chemotherapy from the adjusted model is computed, and a new benchmark population is formed as patients from hospitals with top 10% average of the predicted probabilities. To estimate the perioperative chemotherapy rates and its confidence intervals for the new benchmark and nonbenchmark populations, we used a parametric bootstrapping approach with 1000 bootstrap samples to simulate perioperative chemotherapy use using the predicted probability of each patient in the populations. The perioperative chemotherapy rate and its 95% CI are obtained as the average and the 2.5th and 97.5th percentiles from 1000 bootstrap samples of perioperative chemotherapy use in each population. Differences in hospital perioperative chemotherapy rates were compared using logistic and modified Poisson regression with the hospital as a fixed effect. We also calculated the intraclass correlation coefficient (ICC) to determine the degree of variation in perioperative CT rates between hospitals compared to the variation within hospitals.^26^

Finally, to explore the extent to which our results were sensitive to sample size, we repeated the above analysis using geographic region instead of hospital as the unit of analysis. Statistical analyses were carried out using SAS version 9.4 (SAS Institute). The study was approved by the Research Ethics Board of Queen's University, Kingston, Canada.

## 3 RESULTS

### 3.1 Study population

The study population included 2581 patients (Supplemental Figure S1 and Table 1). Fifty percent (1301/2581) of patients had surgery at a teaching hospital; 37% (954/2581) had surgery at a comprehensive cancer centre. Sixty percent (1540/2581) had surgery at a hospital with on‐site medical oncology services.

**Table 1:** Characteristics of patients with muscle‐invasive bladder cancer treated with cystectomy in Ontario 2004‐2013

	All patients	No perioperative chemotherapy	Perioperative chemotherapy	*P* value
	N = 2581	N = 1783	N = 798	
*Patient‐related*				
Year of Surgery				<.001
2004‐2008	1275 (49%)	936 (52%)	339 (42%)	
2009‐2013	1306 (51%)	847 (48%)	459 (58%)	
Age (years)				<.001
20‐49	72 (3%)	25 (1%)	47 (6%)	
50‐59	322 (12%)	152 (9%)	170 (21%)	
60‐69	665 (26%)	395 (22%)	270 (34%)	
70‐79	995 (39%)	730 (41%)	265 (33%)	
80+	527 (20%)	481 (27%)	46 (6%)	
Sex				.443
Female	620 (24%)	436 (24%)	184 (23%)	
Male	1961 (76%)	1347 (76%)	614 (77%)	
SES by quintile^a^				.263
1	498 (19%)	362 (20%)	136 (17%)	
2	589 (23%)	403 (23%)	186 (23%)	
3	570 (22%)	401 (22%)	169 (21%)	
4	462 (18%)	311 (17%)	151 (19%)	
5	424 (16%)	279 (16%)	145 (18%)	
Unknown	38 (1%)	27 (2%)	11 (1%)	
Charlson comorbidity score				<.001
0	1774 (69%)	1165 (65%)	609 (76%)	
1+	807 (31%)	618 (35%)	189 (24%)	
Rural status^b^				.379
No	2170‐2175 (84%)	1502‐1507 (84%)	665‐670 (84%)	
Yes	404 (16%)	276 (15%)	128 (16%)	
Unknown	≤5 (0%)	≤5 (0%)	≤5 (0%)	
Region^c^				.033
A	149 (6%)	103 (6%)	46 (6%)	
B	211 (8%)	133 (7%)	78 (10%)	
C	154 (6%)	123 (7%)	31 (4%)	
D	321 (12%)	232 (13%)	89 (11%)	
E	121 (5%)	81 (5%)	40 (5%)	
F	190 (7%)	122 (7%)	68 (9%)	
G	187 (7%)	130 (7%)	57 (7%)	
H	282 (11%)	188 (11%)	94 (12%)	
I	310 (12%)	221 (12%)	89 (11%)	
J	126 (5%)	82 (5%)	44 (6%)	
K	201 (8%)	145 (8%)	56 (7%)	
L	122 (5%)	75 (4%)	47 (6%)	
M	163 (6%)	117 (7%)	46 (6%)	
N	40‐45 (2%)	27‐31 (2%)	8‐13 (1%)	
Unknown	≤5 (0%)	≤5 (0%)	≤5 (0%)	
*Disease‐related* d				
T stage				<.001
<T3	642‐648 (25%)	549 (31%)	94‐99 (12%)	
T3‐T4	1620 (63%)	1234 (69%)	386 (48%)	
Unstated	≤5 (0%)	0 (0%)	≤5 (0%)	
Unreported	314 (12%)	0 (0%)	314 (39%)	
N stage				<.001
N0	1276 (49%)	1149 (64%)	127 (16%)	
N1+	773 (30%)	441 (25%)	332 (42%)	
NX	218 (8%)	193 (11%)	25 (3%)	
Unreported	314 (12%)	0 (0%)	314 (39%)	
LVI				<.001
No	739 (29%)	641 (36%)	98 (12%)	
Yes	1226 (48%)	896 (50%)	330 (41%)	
Unstated	302 (12%)	246 (14%)	56 (7%)	
Unreported	314 (12%)	0 (0%)	314 (39%)	
Pelvic lymph node count				<.001
Mean/Median	12/10	11/10	13/11	
≤13	1342 (52%)	935 (52%)	407 (51%)	
>13	738 (29%)	444 (25%)	294 (37%)	
Not accessed	501 (19%)	404 (23%)	97 (12%)	
*Cystectomy hospital‐related*				
Teaching hospital				<.001
No	1280 (50%)	930 (52%)	350 (44%)	
Yes	1301 (50%)	853 (48%)	448 (56%)	
Medical oncologist onsite				.673
No	1041 (40%)	724 (41%)	317 (40%)	
Yes	1540 (60%)	1059 (59%)	481 (60%)	
Regional cancer center				.052
No	1627 (63%)	1146 (64%)	481 (60%)	
Yes	954 (37%)	637 (36%)	317 (40%)	
Surgeon volume, quartile^e^				<.001
Q1	561 (22%)	400 (22%)	161 (20%)	
Q2	686 (27%)	461 (26%)	225 (28%)	
Q3	643 (25%)	479 (27%)	164 (21%)	
Q4	685‐690 (27%)	440‐445 (25%)	245‐250 (31%)	
Unknown	≤5 (0%)	≤5 (0%)	≤5 (0%)	
Hospital volume, quartile^e^				.002
Q1	556 (22%)	392 (22%)	164 (21%)	
Q2	746 (29%)	531 (30%)	215 (27%)	
Q3	614 (24%)	440 (25%)	174 (22%)	
Q4	665 (26%)	420 (24%)	245 (31%)	

Note

As per Institute of Clinical Evaluative Sciences policy, cells were suppressed to ensure that precise small cell values cannot be determined.

Abbreviations: SES, socioeconomic status, LVI, lymphovascular invasion.

a Socioeconomic status, Quintile 1 represents communities where the poorest 20% of the Ontario population resided. SES data were not available for 38 patients.

b Rural status is assigned if a residence postal code is found in a community with <10 000 people. Rural status data were not available for ≤5 patients.

c Region data were not available for ≤5 patients.

d Pathologic T stage, N stage, and LVI are only reported for cases that did not receive NACT. T stage was unstated for ≤5 patients.

e Surgeon and hospital volume quartile 1 represent the lowest surgeon and hospital volumes. Surgeon volume data were unavailable for ≤5 patients.

### 3.2 Interhospital and interregional variation in ACT rates

**Figure 1:**
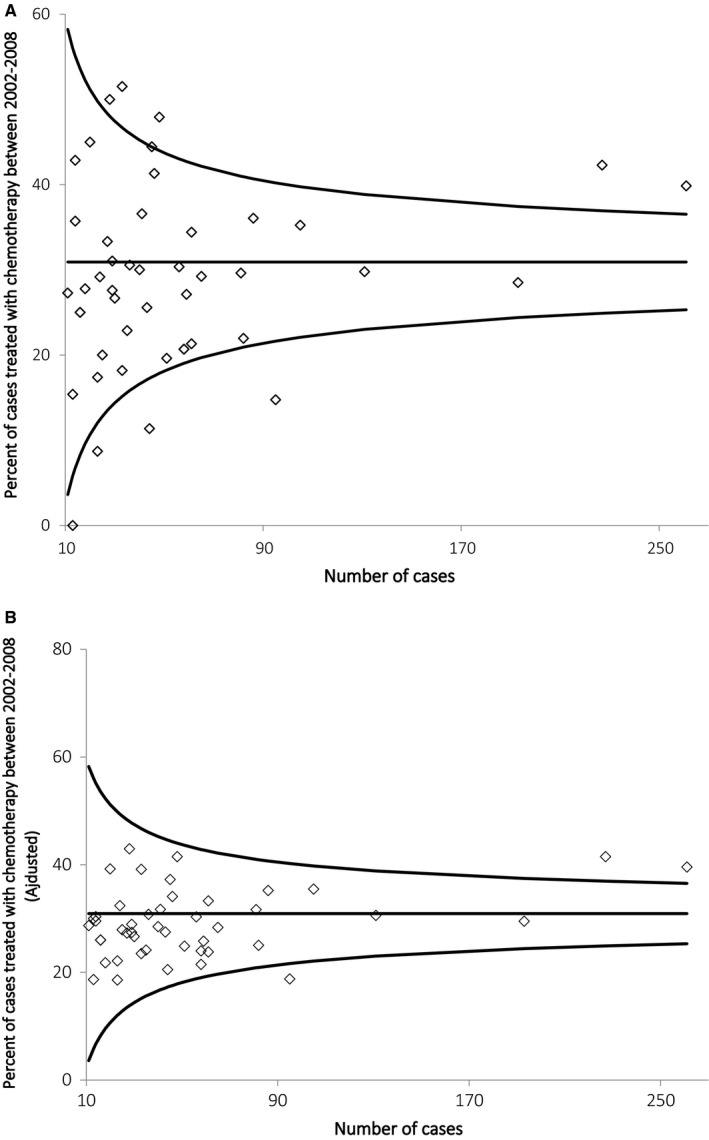
(A) Inter‐hospital variation in the use of perioperative chemotherapy for muscle‐invasive bladder cancer in Ontario 2004‐2013. The provincial mean rate is the horizontal line, 95% confidence intervals are shown with the additional lines. (B) Interhospital variation in the adjusted perioperative chemotherapy rate for muscle‐invasive bladder cancer in Ontario 2004‐2013. The provincial mean rate is the horizontal line, 95% confidence intervals are shown with the additional lines. Hospitals with volumes less than 10 were removed from the figure (N = 46 hospitals)

Perioperative chemotherapy was delivered to 31% (798/2581) of the population; treatment rates were higher during 2009‐2013 compared to 2004‐2008 (35% vs 27%, *P* < .001). After excluding 34 patients with surgery at hospitals treating <10 cases, hospital‐specific CT rates varied from 0% to 52% (IQR 21%‐36%) (Figure 1A). Figure 1B shows the hospital‐specific rates after adjusting for patient and disease‐specific factors that may influence the use of perioperative chemotherapy. The observed CV of the hospital‐specific perioperative CT rates was 37.9%. At several individual hospitals the observed perioperative CT rate fell outside the 95% CI of the province‐wide rate (Figure 1A and B). Furthermore, results of the Monte Carlo simulation showed that if the underlying probability was identical to the provincial rate, chance alone would not lead to a similar degree of variation in hospital‐specific perioperative CT rates, with an expected CV = 12.7% (95% CI 9.71%‐16.10%). Thus, the observed CV is much higher than could be observed by chance alone. When this analysis was repeated by region, the CV of the region‐specific specific perioperative chemotherapy rates was 23.7% and the expected CV was 6.6% (95% CI 4.05%‐9.94%); this signifies that the variation in practice between regions is substantially different from variation due to chance alone.

### 3.3 CBB: System‐level factors associated with the use of perioperative chemotherapy

Criterion‐based benchmarks are derived from the rates of treatment in a benchmark population of patients that represents those who are managed in a setting that supports optimal decision making and who have optimal access to treatment. We hypothesized that decision making about perioperative chemotherapy for MIBC was most likely to be optimal in teaching hospitals, in comprehensive cancer centres and hospitals where medical oncologists were available on‐site. In addition, we predicted that patients who resided in neighborhoods with a higher SES would have greater access to treatment. We tested these hypotheses using multivariate logistic regression that explored the impact of these system‐related and socioeconomic factors on use of chemotherapy (Table 2). As stage is only reliable in patients who did not receive NACT, we could not adjust for this variable. Residents of richer communities (*P* = .033) and those who had their cystectomy at a teaching hospital (*P* = .002) were more likely to receive perioperative chemotherapy. We did not find any measurable difference in chemotherapy rates by comprehensive cancer centre status, or having medical oncology on site.

**Table 2:** Factors associated with perioperative chemotherapy utilization among 2581 patients with muscle invasive bladder cancer treated with cystectomy in Ontario 2004‐2013

Characteristic	% Any chemo (unadjusted)	Univariate analysis		% Any chemo (adjusted)	Multivariate analysis	
		RR (95% CI)	*P* value		RR (95% CI)	*P* value
Age, years			<.001			<.001
20‐49	65	Ref		63	Ref	
50‐59	53	0.81 (0.66‐0.99)		51	0.82 (0.68‐0.99)	
60‐69	41	0.62 (0.51‐0.75)		40	0.66 (0.55‐0.79)	
70‐79	27	0.41 (0.33‐0.50)		27	0.43 (0.36‐0.52)	
80+	9	0.13 (0.10‐0.18)		9	0.14 (0.10‐0.20)	
Sex			.440			.854
Female	30	0.95 (0.83‐1.09)		28	0.99 (0.87‐1.13)	
Male	31	Ref		28	Ref	
SES^a^, quintile			.167			.033
Q1	27	Ref		23	Ref	
Q2	32	1.16 (0.96‐1.39)		28	1.19 (1.00‐1.43)	
Q3	30	1.09 (0.90‐1.31)		27	1.15 (0.96‐1.38)	
Q4	33	1.20 (0.99‐1.45)		29	1.22 (1.01‐1.47)	
Q5	34	1.25 (1.03‐1.52)		33	1.34 (1.12‐1.62)	
Charlson comorbidity score			<.001			<.001
0	34	Ref		31	Ref	
1+	23	0.68 (0.59‐0.79)		23	0.77 (0.67‐0.88)	
Teaching hospital			<.001			.002
No	27	Ref		26	Ref	
Yes	34	1.26 (1.12‐1.42)		31	1.19 (1.06‐1.33)	

a Socioeconomic status, Quintile 1 represents communities where the poorest 20% of the Ontario population resided. SES data were not available for 38 patients.

Based on these results, we defined the benchmark population as patients who resided in a high‐income community (SES Q5 community) and those that had their cystectomy at a teaching hospital. As shown in Table 3, characteristics of the benchmark population were otherwise similar to the nonbenchmark population. The benchmark perioperative chemotherapy rate was 36% (95% CI 30%‐44%) compared to 31% (95% CI 29%‐33%) observed in the overall study population; the relative shortfall in utilization is therefore 13.9%.

**Table 3:** Characteristics of the benchmark (defined by highest SES quintile and cystectomy performed at teaching hospital using the criterion‐based benchmarking approach) and nonbenchmark populations among patients with muscle‐invasive bladder cancer treated with cystectomy in Ontario 2004‐2013

	All patients	Benchmark population	Nonbenchmark population	*P* value
	N = 2581	N = 195	N = 2386	
*Patient‐related*				
Year of Surgery				.165
2004‐2008	1275 (49%)	87 (45%)	1188 (50%)	
2009‐2013	1306 (51%)	108 (55%)	1198 (50%)	
Age (years)				.294
20‐49	72 (3%)	7 (4%)	65 (3%)	
50‐59	322 (12%)	18 (9%)	304 (13%)	
60‐69	665 (26%)	59 (30%)	606 (25%)	
70‐79	995 (39%)	68 (35%)	927 (39%)	
80+	527 (20%)	43 (22%)	484 (20%)	
Sex				.086
Female	620 (24%)	37 (19%)	583 (24%)	
Male	1961 (76%)	158 (81%)	1803 (76%)	
SES by quintile^a^				<.001
1	498 (19%)	–	498 (21%)	
2	589 (23%)	–	589 (25%)	
3	570 (22%)	–	570 (24%)	
4	462 (18%)	–	462 (19%)	
5	424 (16%)	195 (100%)	229 (10%)	
Unknown	38 (1%)	0 (0%)	38 (2%)	
Charlson comorbidity score				
0	1774 (69%)	134 (69%)	1640 (69%)	.996
1+	807 (31%)	61 (31%)	746 (31%)	
*System‐related*				
Teach				
No	1280 (50%)	–	1280 (54%)	<.001
Yes	1301 (50%)	195 (100%)	1106 (46%)	
Pelvic lymph node count				<.001
Mean/Median	12/10	14/13	12/10	
≤13	1342 (52%)	102 (52%)	1240 (52%)	
>13	738 (29%)	80 (41%)	658 (28%)	
Not accessed	501 (19%)	13 (7%)	488 (20%)	
*Treatment‐related*				
Any chemotherapy				
No	1783 (69%)	124 (64%)	1659 (70%)	.084
Yes	798 (31%)	71 (36%)	727 (30%)	
Surgeon volume, quartile^b^				<.001
Q1	561 (22%)	8 (4%)	553 (23%)	
Q2	686 (27%)	29 (15%)	657 (28%)	
Q3	643 (25%)	56 (29%)	587 (25%)	
Q4	685‐690 (27%)	102 (52%)	582‐587 (25%)	
Unknown	≤5 (0%)	0 (0%)	≤5 (0%)	
Hospital volume, quartile^b^				<.001
Q1	556 (22%)	14 (7%)	542 (23%)	
Q2	746 (29%)	13 (7%)	733 (31%)	
Q3	614 (24%)	51 (26%)	563 (24%)	
Q4	665 (26%)	117 (60%)	548 (23%)	

Note

As per Institute of Clinical Evaluative Sciences policy, cells were suppressed to ensure that precise small cell values cannot be determined.

Abbreviation: SES, socioeconomic status.

a Socioeconomic status, Quintile 1 represents communities where the poorest 20% of the Ontario population resided. SES data were not available for 38 patients.

b Surgeon and hospital volume quartile 1 represent the lowest surgeon and hospital volumes**.** Surgeon volume data were unavailable for ≤ 5 patients.

### 3.4 ABC method

**Figure 2:**
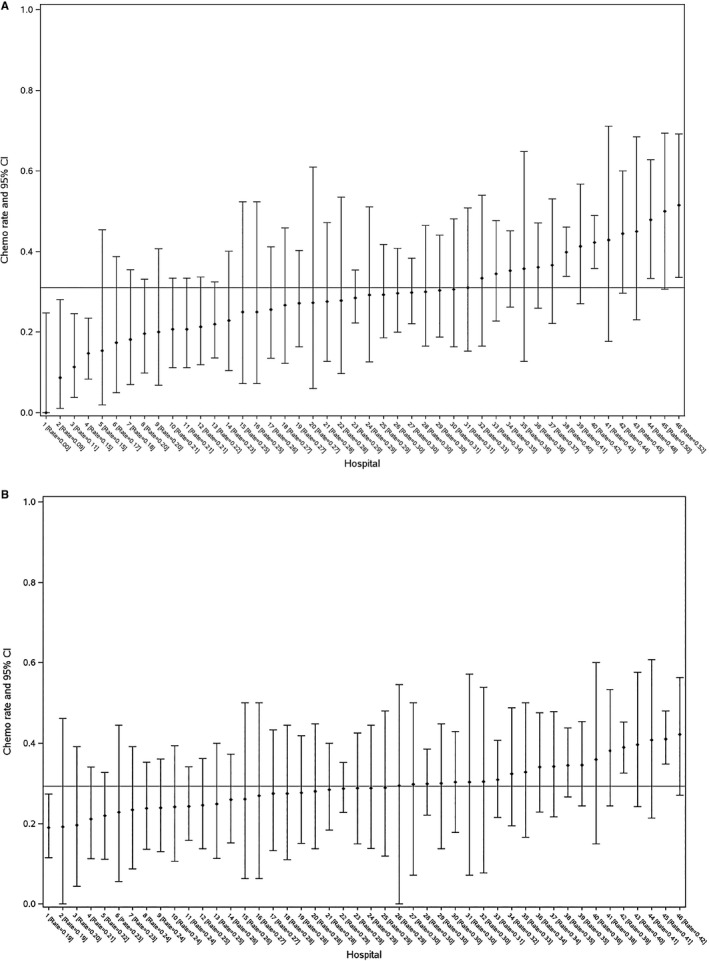
Unadjusted (Panel A) and adjusted (Panel B*) hospital utilization rates of perioperative chemotherapy for 2547 patients MIBC treated in Ontario during 2004‐2013. *Covariates adjusted for at the level of the patient include: age, sex, and ses and charlson comorbidity. Rates of perioperative chemotherapy and 95% CIs were obtained using a parametric bootstrapping approach consisting of 1000 simulations of the predicted probabilities for each patient from the multivariable regression model

After removing patients from hospitals with <10 cases (n = 34 cases), we applied the ABC method to 2547 patients. Figure 2 lists hospitals in ascending order of perioperative CT utilization rates. There were 415 patients in the seven benchmark hospitals. Patient characteristics of the benchmark and nonbenchmark populations were largely comparable; however, the nonbenchmark population had lower volume providers and fewer lymph node dissections (Table 4). The unadjusted perioperative chemotherapy rate in the benchmark population was 45% (95% CI 40%‐50%) vs 28% (95% CI 26%‐30%) in the nonbenchmark population, and 31% in the province as a whole. If the rate of perioperative chemotherapy in the benchmark population was accepted as the optimal rate, the percentage shortfall in the use of chemotherapy was calculated as [(45%‐31%)/45% × 100%] suggesting that 31.1% of the patients who should have received chemotherapy did not receive it.

**Table 4:** Characteristics of patients with muscle‐invasive bladder cancer treated in Ontario during 2004‐2013 classified by hospital benchmark status using the ABC method

Characteristic	All patients	Nonbenchmark population	Benchmark population	*P*‐value
	N = 2547	N = 2132	N = 415	
*Patient‐related*				
Year of Surgery				.643
2004‐2008	1254 (49%)	1054 (49%)	200 (48%)	
2009‐2013	1293 (51%)	1078 (51%)	215 (52%)	
Age, in years				.020
20‐49	72 (3%)	54 (3%)	18 (4%)	
50‐59	319 (13%)	253 (12%)	66 (16%)	
60‐69	659 (26%)	548 (26%)	111 (27%)	
70‐79	979 (38%)	838 (39%)	141 (34%)	
80+	518 (20%)	439 (21%)	79 (19%)	
Sex				.124
Female	609 (24%)	522 (24%)	87 (21%)	
Male	1938 (76%)	1610 (76%)	328 (79%)	
SES, by quintile^a^				<.001
1	486 (19%)	420 (20%)	66 (16%)	
2	583 (23%)	502 (24%)	81 (20%)	
3	566 (22%)	481 (23%)	85 (20%)	
4	457 (18%)	389 (18%)	68 (16%)	
5	417 (16%)	310 (15%)	107 (26%)	
Unknown	38 (1%)	30 (1%)	8 (2%)	
Rural Status^b^				.746
No	2146 (84%)	1796 (84%)	350 (84%)	
Yes	395‐400 (16%)	330‐335 (16%)	65 (16%)	
Unknown	≤5 (0%)	≤5 (0%)	0 (0%)	
Charlson comorbidity score				.790
0	1751 (69%)	1468 (69%)	283 (68%)	
1+	796 (31%)	664 (31%)	132 (32%)	
*Treatment‐related*				
Perioperative chemotherapy				<.001
No	1758 (69%)	1528 (72%)	230 (55%)	
Yes	789 (31%)	604 (28%)	185 (45%)	
Pelvic lymph node count				<.001
Mean/Median	12/10	12/10	13/11	
≤13	1326 (52%)	1108 (52%)	218 (53%)	
>13	735 (29%)	577 (27%)	158 (38%)	
Not accessed	486 (19%)	447 (21%)	39 (9%)	
*System‐related*				
Teaching hospital				<.001
No	1246 (49%)	1106 (52%)	140 (34%)	
Yes	1301 (51%)	1026 (48%)	275 (66%)	
Surgeon volume, quartile^c^				<.001
Q1	531 (21%)	476 (22%)	55 (13%)	
Q2	682 (27%)	508 (24%)	174 (42%)	
Q3	643 (25%)	578 (27%)	65 (16%)	
Q4	685‐690 (27%)	565‐570 (27%)	115‐120 (29%)	
Unknown	≤5 (0%)	≤5 (0%)	≤5 (0%)	
Hospital volume, quartile^c^				<.001
Q1	528 (21%)	429 (20%)	99 (24%)	
Q2	740 (29%)	651 (31%)	89 (21%)	
Q3	614 (24%)	614 (29%)	0 (0%)	
Q4	665 (26%)	438 (21%)	227 (55%)	

Note

As per Institute of Clinical Evaluative Sciences policy, cells were suppressed to ensure that precise small cell values cannot be determined.

Abbreviation: SES, socioeconomic status.

a Socioeconomic status, Quintile 1 represents communities where the poorest 20% of the Ontario population resided. SES data were not available for 38 patients.

b Rural status is assigned if a residence postal code is found in a community with < 10 000 people. Rural status data were not available for ≤ 5 patients.

c Surgeon and hospital volume quartile 1 represent the lowest surgeon and hospital volumes**.** Surgeon volume data were unavailable for ≤ 5 patients.

Subsequently, we adjusted treatment rates by hospital for patient‐related factors using the predicted probability for each patient and a parametric bootstrapping approach to identify an “adjusted hospital benchmark population.” By controlling for differences in case mix across hospitals this would be expected to reduce some of the apparently random variation in the use of chemotherapy. The new benchmark population consisted of 275 patients from two hospitals (Supplemental Table S1). The simulated perioperative CT rate in this benchmark population was 41% (95% CI 35%‐47%) vs 30% (95% CI 28%‐32%) in the nonbenchmark population. If this adjusted chemotherapy rate in the benchmark population was taken as the optimal rate, the shortfall in utilization would be 24.3%. The ICC in the unadjusted and adjusted models was 0.038 and 0.037 respectively, indicating that treatment variation within hospitals is greater than the variation between hospitals.

We repeated the above analysis using geographic region instead of hospital as the unit of analysis. The perioperative chemotherapy rate in the adjusted benchmark population was 37% (95% CI 32%‐43%) vs 30% (95% CI 28%‐32%) in the nonbenchmark population; using this benchmark population rate as the optimal rate, the shortfall in perioperative CT utilization would be 16.2% (Supplemental Appendix).

Given that perioperative CT rates in our cohort had increased significantly between 2004‐2008 and 2009‐2013, we conducted an exploratory analysis to determine a benchmark rate if the analysis was restricted to 2011‐2013. Due to inadequate sample size, this analysis could only be conducted by geographic region and not by hospital. The adjusted benchmark population consisted of 89 patients in one region (Supplementary Table S4). The adjusted perioperative CT rate in the benchmark population was 51% (95% CI 40%‐61%) compared to 37% (95% CI 34%‐41%) in the nonbenchmark population and 38% in the overall population. The calculated shortfall in perioperative CT utilization in 2011‐2013 was 25.5%.

## 4 DISCUSSION

In this study, we report estimates of a benchmark utilization rate for perioperative chemotherapy in MIBC. Several important findings have emerged. First, patients who resided in a high‐income community and those who had surgery at a teaching hospital were more likely to receive chemotherapy. Using the CBB approach and eliminating these system‐level and social barriers we were able to identify a benchmark rate for chemotherapy utilization. Second, using the ABC approach we were able to identify a benchmark rate for perioperative chemotherapy utilization; this rate was different depending on whether hospital or region was used at the unit of analysis. The benchmark rate from these various approaches is 36%‐41%. If the analysis was restricted to the most recent 2 years, the benchmark rate was 51%. Third, the results of the Monte Carlo simulation suggest that it is unlikely that the observed variation in chemotherapy rates is due to chance alone.

CBB has been widely used to estimate the need for radiotherapy and is used by policy‐makers to measure health system performance.^18^ MacKillop et al used this approach to estimate a benchmark rate for palliative radiation therapy (RT) in Ontario.^27^ In their analysis, they found that patients whose cancer was diagnosed with RT on site, residents of richer communities and those who lived closer to an RT facility were more likely to receive RT. These factors were used to determine a benchmark and nonbenchmark rate of 34% and 26%, respectively; yielding a shortfall in RT utilization of 16%. In our study, patients from higher income communities and those who had surgery at a teaching hospital represented the benchmark population. Using this approach, we estimated that the optimal rate of perioperative chemotherapy utilization for MIBC during 2004‐2013 was 36% and 14% of patients who should have received this treatment, did not.

We used the ABC method to estimate a benchmark rate for perioperative chemotherapy and used both hospital and region as the unit of analysis. When conducting the analysis by hospital, we found an unadjusted and adjusted benchmark rate of 45% and 41%. When using region as the unit of analysis, unadjusted and adjusted benchmark rates were 39% and 37%.

Our analysis showed that when we restricted our cohort to those who received cystectomy for MIBC between 2011 and 2013, the adjusted benchmark rate using region was 51% compared to 37% from 2004 to 2013. Several studies have shown that the uptake of perioperative treatment for MIBC has been increasing in recent years^10^, ^11^, ^12^; the increase in benchmark rates in the most recent 2 years of our analysis confirms these findings. Consequently, if current utilization rates are similar to those seen in 2011‐2013, the benchmark rate derived from these 2 years may be a more accurate estimate of an optimal rate for which to benchmark current practice.

We found that there was wide variation in chemotherapy rates across institutions; there was a 23 point spread in utilization rates between the highest and the lowest ranked hospitals (42% vs 19%). However, the ICC value of 0.037 in the adjusted hospital model signifies that there is a large degree of variation in practice within individual hospitals; this may reflect varied preferences of individual urologists and medical oncologists.

An important aspect of our analysis was to determine whether the observed variation between hospitals could be caused by chance alone. The results of the Monte Carlo simulations showed that the observed CV fell outside of the 95% CI of the simulation results, implying the variation is unlikely due to random chance. This is in contrast to what we observed in our analysis of estimating a benchmark rate for ACT in stage III colon cancer (see companion paper); while there was a difference between the benchmark and nonbenchmark rates of ACT usage, the simulation results showed that this was likely the result of chance. These findings may be explained by the fact that while ACT for stage III colon cancer has been widely accepted for many years, perioperative chemotherapy for MIBC is a relatively new practice and therefore wide variation in its use may be observed in practice. Future studies that adopt the ABC method should consider whether the observed variation could be due to random chance.

Our study has limitations which warrant comment. As the stage of cancer is only reliable in patients who did not received NACT, we were not able to control for disease‐related factors in our regression analysis. However, we found that the distribution of T stage and N stage was relatively consistent across regions (data not shown), thereby limiting the potential confounding effect in our analysis. Secondly, detailed information related to patient preferences, comorbidity, laboratory data, and performance status is not available; this limits our ability to evaluate the appropriateness of case selection for chemotherapy.

In summary, we have derived an estimate benchmark rate for perioperative chemotherapy for MIBC in the province of Ontario using two different approaches. We estimate that the benchmark rate is 36%‐41% and may be higher in more recent years. Unlike our benchmarking approach for ACT in stage III colon cancer, we conclude that a benchmark rate can be accurately estimated for perioperative chemotherapy for MIBC and therefore could be used to drive improvements in quality of care.

## CONFLICT OF INTEREST

The authors report no conflict of interest.

## Data Availability

The data that support the findings of this study are available from the Institute for Clinical Evaluative Sciences. Restrictions apply to the availability of these data, which were used under license for this study.
