# Supplementary material for: Estimating the optimal perioperative chemotherapy utilization rate for muscle‐invasive bladder cancer
Source: Cancer Med. 2019 Aug 31;8(14):6258–71. doi: 10.1002/cam4.2449 (PMC6797575; doi:10.1002/cam4.2449)
Supplement: Supplementary file 1 [file CAM4-8-6258-s001.pdf]

Supplemental eFigure1. Identification of patients with muscle-invasive bladder cancer treated with cystectomy in Ontario 2004-2013

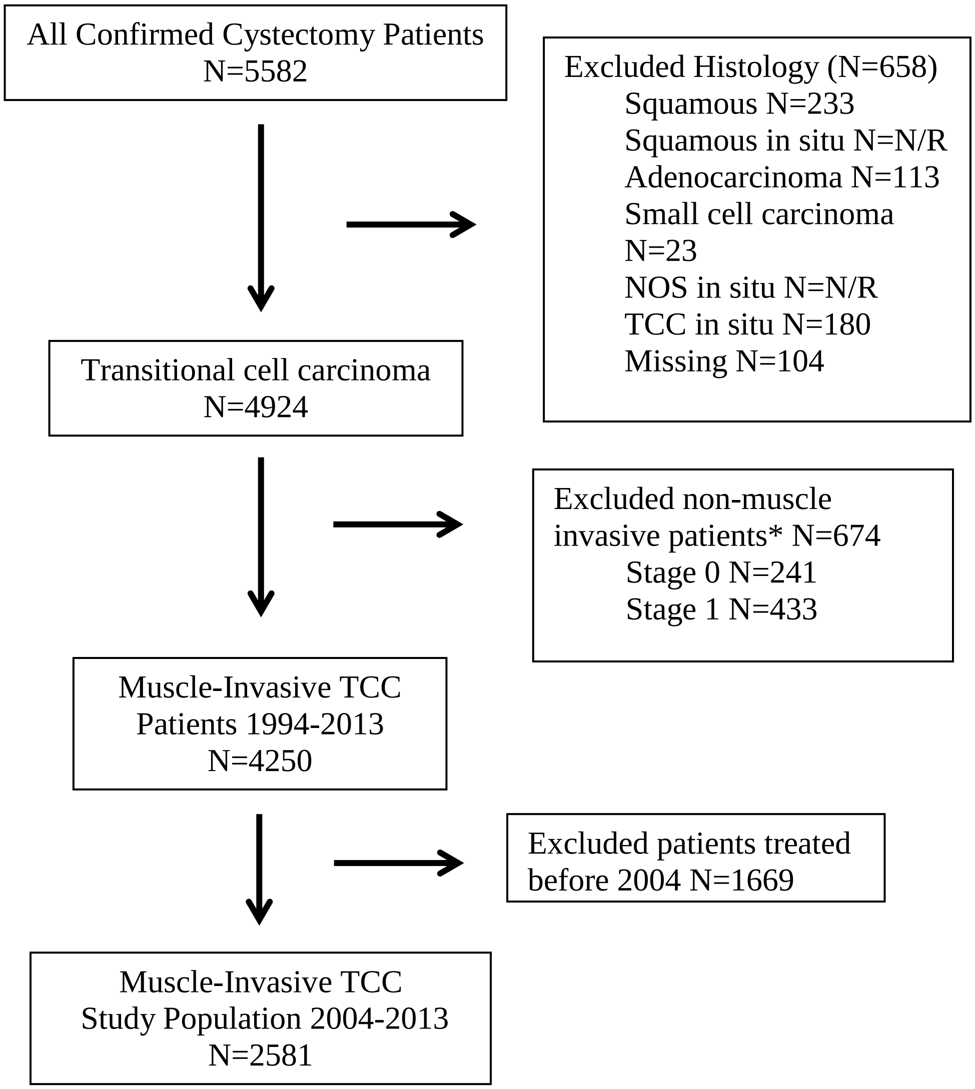

***Note**: Stage 0/1 patients that were treated with pre-operative RT (n=1) and/or NACT (n=84) and/or had nodal involvement (n=17) were not excluded from the muscle-invasive study population.

N/R: Note due to small cell size
NOS: Not otherwise specified
