# Supplementary material for: Estimating the optimal perioperative chemotherapy utilization rate for muscle‐invasive bladder cancer
Source: Cancer Med. 2019 Aug 31;8(14):6258–71. doi: 10.1002/cam4.2449 (PMC6797575; doi:10.1002/cam4.2449)
Supplement: Supplementary file 2 [file CAM4-8-6258-s002.pdf]

Supplemental eFigure 2. Unadjusted (Panel A) and adjusted (Panel B) regional utilization rates of perioperative chemotherapy for 2581 patients MIBC treated in Ontario during 2004-2013.

A

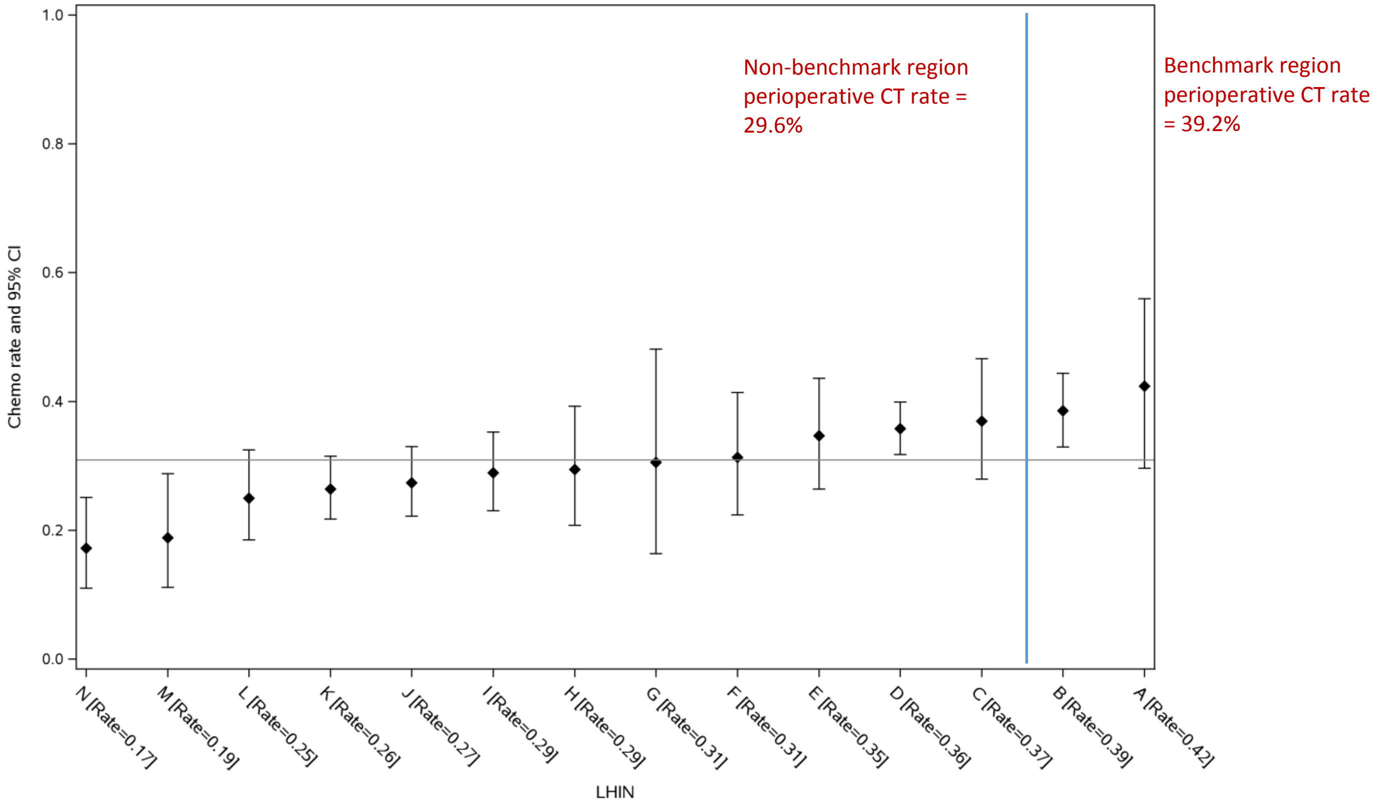

B

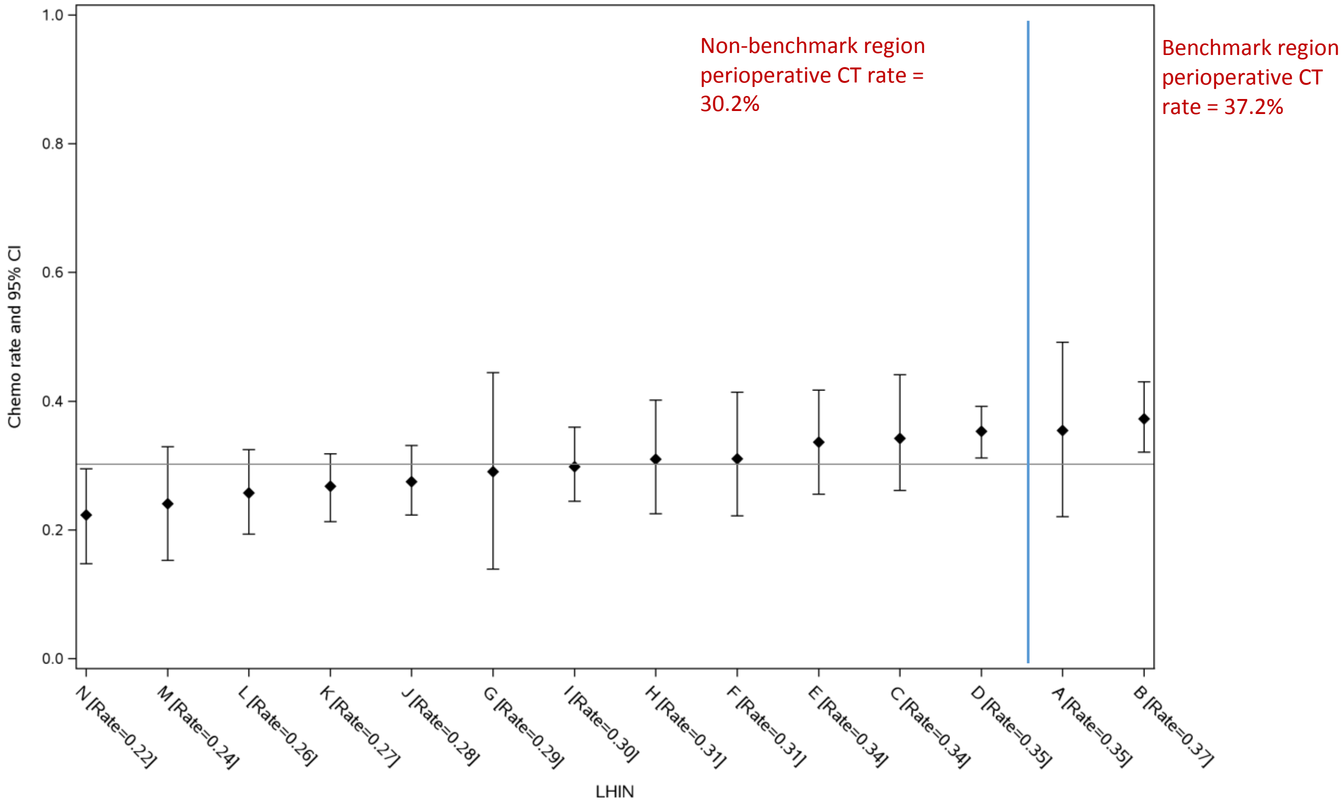

*Covariates adjusted for at the level of the patient include: age, sex, and ses and charlson comorbidity. Rates of perioperative chemotherapy and 95% CIs were obtained using a parametric bootstrapping approach consisting of 1000 simulations of the predicted probabilities for each patient from the multivariable regression model.
