# Supplementary material for: Estimating the optimal perioperative chemotherapy utilization rate for muscle‐invasive bladder cancer
Source: Cancer Med. 2019 Aug 31;8(14):6258–71. doi: 10.1002/cam4.2449 (PMC6797575; doi:10.1002/cam4.2449)
Supplement: Supplementary file 3 [file CAM4-8-6258-s003.docx]

**Supplemental Table S1.** Characteristics of patients with muscle-invasive bladder cancer treated in Ontario during 2004-2013 classified by hospital benchmark status after case mix adjustment

| **Characteristic** | **All patients** | **Non-benchmark population** | **Benchmark population** | **p-value** |
| --- | --- | --- | --- | --- |
|  | N=2,547 | N=2,272 | N=275 |  |
| ***Patient-related*** |  |  |  |  |
| Year of Surgery |  |  |  | 0.491 |
| 2004-2008 | 1,254 (49%) | 1,124 (49%) | 130 (47%) |  |
| 2009-2013 | 1,293 (51%) | 1,148 (51%) | 145 (53%) |  |
| Age, in years |  |  |  | 0.031 |
| 20-49 | 72 (3%) | 58 (3%) | 14 (5%) |  |
| 50-59 | 319 (13%) | 275 (12%) | 44 (16%) |  |
| 60-69 | 659 (26%) | 587 (26%) | 72 (26%) |  |
| 70-79 | 979 (38%) | 882 (39%) | 97 (35%) |  |
| 80+ | 518 (20%) | 470 (21%) | 48 (17%) |  |
| Sex |  |  |  | 0.107 |
| Female | 609 (24%) | 554 (24%) | 55 (20%) |  |
| Male | 1,938 (76%) | 1,718 (76%) | 220 (80%) |  |
| SES, by quintile^1^ |  |  |  | <0.001 |
| 1 | 486 (19%) | 443 (19%) | 43 (16%) |  |
| 2 | 583 (23%) | 531 (23%) | 52 (19%) |  |
| 3 | 566 (22%) | 525 (23%) | 41 (15%) |  |
| 4 | 457 (18%) | 410 (18%) | 47 (17%) |  |
| 5 | 417 (16%) | 332 (15%) | 85 (31%) |  |
| Unknown | 38 (1%) | 31 (1%) | 7 (3%) |  |
| Rural Status^2^ |  |  |  | 0.820 |
| No | 2,146 (84%) | 1,913 (84%) | 233 (85%) |  |
| Yes | 395-400 (16%) | 355-360 (16%) | 42 (15%) |  |
| Unknown | ≤5 (0%) | ≤5 (0%) | 0 (0%) |  |
| Charlson comorbidity score |  |  |  | 0.212 |
| 0 | 1,751 (69%) | 1,571 (69%) | 180 (65%) |  |
| 1+ | 796 (31%) | 701 (31%) | 95 (35%) |  |
| ***Treatment-related*** |  |  |  | <0.001 |
| Pelvic lymph node count |  |  |  |  |
| Mean/Median | 12/10 | 12/10 | 14/13 |  |
| ≤13 | 1,326 (52%) | 1,184 (52%) | 142 (52%) |  |
| >13 | 735 (29%) | 612 (27%) | 123 (45%) |  |
| Not accessed | 486 (19%) | 476 (21%) | 10 (4%) |  |

| ***System-related*** |  |  |  |  |
| --- | --- | --- | --- | --- |
| Teaching hospital |  |  |  | <0.001 |
| No | 1,246 (49%) | 1,198 (53%) | 48 (17%) |  |
| Yes | 1,301 (51%) | 1,074 (47%) | 227 (83%) |  |
| Surgeon volume, quartile^3^ |  |  |  | <0.001 |
| Q1 | 531 (21%) | 515 (23%) | 16 (6%) |  |
| Q2 | 682 (27%) | 596 (26%) | 86 (31%) |  |
| Q3 | 643 (25%) | 590 (26%) | 53 (19%) |  |
| Q4 | 685-690 (27%) | 565-570 (25%) | 120 (44%) |  |
| Unknown | ≤5 (0%) | ≤5 (0%) | 0 (0%) |  |
| Hospital volume, quartile^3^ |  |  |  | <0.001 |
| Q1 | 528 (21%) | 528 (23%) | 0 (0%) |  |
| Q2 | 740 (29%) | 692 (30%) | 48 (17%) |  |
| Q3 | 614 (24%) | 614 (27%) | 0 (0%) |  |
| Q4 | 665 (26%) | 438 (19%) | 227 (83%) |  |

Abbreviations: SES, socioeconomic status.

As per Institute of Clinical Evaluative Sciences policy, cells were suppressed to ensure that precise small cell values cannot be determined.

^1^ Socioeconomic status, Quintile 1 represents communities where the poorest 20% of the Ontario population resided. SES data were not available for 38 patients.

^2^ Rural status is assigned if a residence postal code is found in a community with <10 000 people. Rural status data were not available for ≤5 patients.

^3^ Surgeon and hospital volume quartile 1 represent the lowest surgeon and hospital volumes**.** Surgeon volume data were unavailable for ≤5 patients.

**Appendix: Bladder Chemotherapy Benchmarking Geographic Region Approach**

**METHODS**

To explore the extent to which our results were sensitive to sample size, we repeated the above analysis using geographic region instead of hospital as the unit of analysis. Regional care was described at the level of Ontario’s health regions

**RESULTS**

Supplemental Figure 3 shows the regions in descending order of perioperative CT rates (panel A = before adjustment for case mix, panel B = after adjustment). In the unadjusted model, two regions and 352 patients made up the benchmark population (Supplemental Table 2). More patients in the benchmark population had their cystectomy at a teaching hospital, and at the highest quartile patient and surgical volume hospital (p<0.001). The unadjusted perioperative CT rate in the benchmark population was 39% (95% CI 34%-45%) versus 30% (95% CI 28%-32%) in the non-benchmark population.

The observed CV using the region as the unit of analysis was 23.7%. Results of the Monte Carlo approach showed that the mean simulated CV was 6.6% (95%CI 4.1%-9.9%). The observed CV fell outside the interval of what could be expected due to chance alone, suggesting that differences between region perioperative CT rates are unlikely due to random chance and systematic variation likely exists between regions.

After adjusting for patient- and disease-related characteristics using the parametric bootstrapping approach, we identified an “adjusted region benchmark population” including 293 patients from one region (Supplemental Table 3). The adjusted perioperative CT rate was 37% (95% CI 32%-43%) in the benchmark population vs 30% (95% CI 28%-32%) in the non-benchmark population. These region rates were significantly different when tested as fixed effects using logistic regression and modified Poisson regression (p<0.001). The ICC in the unadjusted and adjusted models was 0.0219 and 0.0209, respectively, indicating that the perioperative CT rate variation within regions is greater than the variation between regions.

**Supplemental Table S2.** Characteristics of patients with MIBC treated in Ontario during 2004-2013 classified by regional benchmark status

| **Characteristic** | **All patients** | **Non-benchmark** | **Benchmark** | **p-value** |
| --- | --- | --- | --- | --- |
|  | N=2,581 | N=2,229 | N=352 |  |
| ***Patient-related*** |  |  |  |  |
| Year of Surgery |  |  |  | 0.083 |
| 2004-2008 | 1,275 (49%) | 1,086 (49%) | 189 (54%) |  |
| 2009-2013 | 1,306 (51%) | 1,143 (51%) | 163 (46%) |  |
| Age, in years |  |  |  | 0.085 |
| 20-49 | 72 (3%) | 58 (3%) | 14 (4%) |  |
| 50-59 | 322 (12%) | 281 (13%) | 41 (12%) |  |
| 60-69 | 665 (26%) | 570 (26%) | 95 (27%) |  |
| 70-79 | 995 (39%) | 848 (38%) | 147 (42%) |  |
| 80+ | 527 (20%) | 472 (21%) | 55 (16%) |  |
| Sex |  |  |  | 0.251 |
| Female | 620 (24%) | 544 (24%) | 76 (22%) |  |
| Male | 1,961 (76%) | 1,685 (76%) | 276 (78%) |  |
| SES, by quintile^1^ |  |  |  | <0.001 |
| 1 | 498 (19%) | 424 (19%) | 74 (21%) |  |
| 2 | 589 (23%) | 457 (21%) | 132 (38%) |  |
| 3 | 570 (22%) | 484 (22%) | 86 (24%) |  |
| 4 | 462 (18%) | 429 (19%) | 33 (9%) |  |
| 5 | 424 (16%) | 399-404 (18%) | 21-26 (7%) |  |
| Unknown | 38 (1%) | 30-35 (2%) | ≤5 (1%) |  |
| Rural Status^2^ |  |  |  | <0.001 |
| No | 2,170-2,175 (84%) | 1,932-1,937 (87%) | 236-241 (68%) |  |
| Yes | 404 (16%) | 293 (13%) | 111 (32%) |  |
| Unknown | ≤5 (0%) | ≤5 (0%) | ≤5 (1%) |  |
| Charlson comorbidity score |  |  |  | 0.810 |
| 0 | 1,774 (69%) | 1,534 (69%) | 240 (68%) |  |
| 1+ | 807 (31%) | 695 (31%) | 112 (32%) |  |
| ***Treatment-related*** |  |  |  |  |
| Peri-operative chemotherapy |  |  |  | <0.001 |
| No | 1,783 (69%) | 1,569 (70%) | 214 (61%) |  |
| Yes | 798 (31%) | 660 (30%) | 138 (39%) |  |

| Pelvic lymph node count |  |  |  | 0.271 |
| --- | --- | --- | --- | --- |
| Mean/Median | 12/10 | 12/10 | 11/10 |  |
| ≤13 | 1,342 (52%) | 1,151 (52%) | 191 (54%) |  |
| >13 | 738 (29%) | 650 (29%) | 88 (25%) |  |
| Not accessed | 501 (19%) | 428 (19%) | 73 (21%) |  |
| ***System-related*** |  |  |  |  |
| Teaching hospital |  |  |  | <0.001 |
| No | 1,280 (50%) | 1,189 (53%) | 91 (26%) |  |
| Yes | 1,301 (50%) | 1,040 (47%) | 261 (74%) |  |
| Surgeon volume, quartile^3^ |  |  |  | <0.001 |
| Q1 | 561 (22%) | 503 (23%) | 58 (16%) |  |
| Q2 | 686 (27%) | 650 (29%) | 36 (10%) |  |
| Q3 | 643 (25%) | 577 (26%) | 66 (19%) |  |
| Q4 | 685-690 (27%) | 493-498 (22%) | 190-195 (54%) |  |
| Unknown | ≤5 (0%) | ≤5 (0%) | ≤5 (0%) |  |
| Hospital volume, quartile^3^ |  |  |  | <0.001 |
| Q1 | 556 (22%) | 501 (22%) | 55 (16%) |  |
| Q2 | 746 (29%) | 710 (32%) | 36 (10%) |  |
| Q3 | 614 (24%) | 614 (28%) | 0 (0%) |  |
| Q4 | 665 (26%) | 404 (18%) | 261 (74%) |  |

Abbreviations: SES, socioeconomic status.

As per Institute of Clinical Evaluative Sciences policy, cells were suppressed to ensure that precise small cell values cannot be determined.

^1^Socioeconomic status, Quintile 1 represents communities where the poorest 20% of the Ontario population resided. SES data were not available for 38 patients.

^2^Rural status is assigned if a residence postal code is found in a community with <10 000 people. Rural status data were not available for ≤5 patients.

^3^Surgeon and hospital volume quartile 1 represent the lowest surgeon and hospital volumes. Surgeon volume data were unavailable for ≤5 patients.

**Supplemental Table S3.** Characteristics of patients with MIBC treated in Ontario during 2004-2013 classified by regional benchmark status after case mix adjustment

| **Characteristic** | **All patients** | **Non-benchmark population** | **Benchmark population** | **p-value** |
| --- | --- | --- | --- | --- |
|  | N=2,581 | N=2,288 | N=293 |  |
| ***Patient-related*** |  |  |  |  |
| Year of Surgery |  |  |  | 0.032 |
| 2004-2008 | 1,275 (49%) | 1,113 (49%) | 162 (55%) |  |
| 2009-2013 | 1,306 (51%) | 1,175 (51%) | 131 (45%) |  |
| Age, in years |  |  |  | 0.070 |
| 20-49 | 72 (3%) | 60 (3%) | 12 (4%) |  |
| 50-59 | 322 (12%) | 288 (13%) | 34 (12%) |  |
| 60-69 | 665 (26%) | 588 (26%) | 77 (26%) |  |
| 70-79 | 995 (39%) | 869 (38%) | 126 (43%) |  |
| 80+ | 527 (20%) | 483 (21%) | 44 (15%) |  |
| Sex |  |  |  | 0.223 |
| Female | 620 (24%) | 558 (24%) | 62 (21%) |  |
| Male | 1,961 (76%) | 1,730 (76%) | 231 (79%) |  |
| SES, by quintile^1^ |  |  |  | <0.001 |
| 1 | 498 (19%) | 436 (19%) | 62 (21%) |  |
| 2 | 589 (23%) | 476 (21%) | 113 (39%) |  |
| 3 | 570 (22%) | 506 (22%) | 64 (22%) |  |
| 4 | 462 (18%) | 433 (19%) | 29 (10%) |  |
| 5 | 424 (16%) | 400-405 (18%) | 20-25 (8%) |  |
| Unknown | 38 (1%) | 30-35 (2%) | ≤5 (1%) |  |
| Rural Status^2^ |  |  |  | <0.001 |
| No | 2,170-2,175 (84%) | 1,970-1,975 (86%) | 198-203 (69%) |  |
| Yes | 404 (16%) | 315 (14%) | 89 (30%) |  |
| Unknown | ≤5 (0%) | ≤5 (0%) | ≤5 (1%) |  |
| Charlson comorbidity score |  |  |  | 0.557 |
| 0 | 1,774 (69%) | 1,577 (69%) | 197 (67%) |  |
| 1+ | 807 (31%) | 711 (31%) | 96 (33%) |  |
| Pelvic lymph node count |  |  |  | 0.948 |
| Mean/Median | 12/10 | 12/10 | 12/11 |  |
| ≤13 | 1,342 (52%) | 1,192 (52%) | 150 (51%) |  |
| >13 | 738 (29%) | 652 (28%) | 86 (29%) |  |
| Not accessed | 501 (19%) | 444 (19%) | 57 (19%) |  |

| ***System-related*** |  |  |  |  |
| --- | --- | --- | --- | --- |
| Teaching hospital |  |  |  | <0.001 |
| No | 1,280 (50%) | 1,248 (55%) | 32 (11%) |  |
| Yes | 1,301 (50%) | 1,040 (45%) | 261 (89%) |  |
| Surgeon volume, quartile^3^ |  |  |  | <0.001 |
| Q1 | 561 (22%) | 535 (23%) | 26 (9%) |  |
| Q2 | 686 (27%) | 665 (29%) | 21 (7%) |  |
| Q3 | 643 (25%) | 589 (26%) | 54 (18%) |  |
| Q4 | 685-690 (27%) | 495-500 (22%) | 190-195 (65%) |  |
| Unknown | ≤5 (0%) | ≤5 (0%) | ≤5 (0%) |  |
| Hospital volume, quartile^3^ |  |  |  | <0.001 |
| Q1 | 556 (22%) | 541 (24%) | 15 (5%) |  |
| Q2 | 746 (29%) | 729 (32%) | 17 (6%) |  |
| Q3 | 614 (24%) | 614 (27%) | 0 (0%) |  |
| Q4 | 665 (26%) | 404 (18%) | 261 (89%) |  |

Abbreviations: SES, socioeconomic status.

As per Institute of Clinical Evaluative Sciences policy, cells were suppressed to ensure that precise small cell values cannot be determined.

^1^Socioeconomic status, Quintile 1 represents communities where the poorest 20% of the Ontario population resided. SES data were not available for 38 patients.

^2^Rural status is assigned if a residence postal code is found in a community with <10 000 people. Rural status data were not available for ≤5 patients.

^3^Surgeon and hospital volume quartile 1 represent the lowest surgeon and hospital volumes. Surgeon volume data were unavailable for ≤5 patients.

**Supplemental Table S4.** Characteristics of patients with MIBC treated in Ontario during 2011-2013 classified by regional benchmark status after case mix adjustment

| **Characteristic** | **All patients** | **Non-benchmark population** | **Benchmark population** | **p-value** |
| --- | --- | --- | --- | --- |
|  | N=786 | N=697 | N=89 |  |
| ***Patient-related*** |  |  |  |  |
| Age, in years |  |  |  | 0.032 |
| 20-49 | 15 (2%) | 11-16 (2%) | ≤5 (≤6%) |  |
| 50-59 | 97 (12%) | 85-90 (12%) | 8-13 (9-15%) |  |
| 60-69 | 215 (27%) | 182 (26%) | 33 (37%) |  |
| 70-79 | 299 (38%) | 262 (38%) | 37 (42%) |  |
| 80+ | 160 (20%) | 152 (22%) | 8 (9%) |  |
| Sex |  |  |  | 0.135 |
| Female | 182 (23%) | 167 (24%) | 15 (17%) |  |
| Male | 604 (77%) | 530 (76%) | 74 (83%) |  |
| SES, by quintile^1^ |  |  |  | 0.005 |
| 1 | 158 (20%) | 135 (19%) | 23 (26%) |  |
| 2 | 175 (22%) | 147 (21%) | 28 (31%) |  |
| 3 | 170 (22%) | 147 (21%) | 23 (26%) |  |
| 4 | 124 (16%) | 117 (17%) | 7 (8%) |  |
| 5 | 132 (17%) | 125-130 (18%) | 6-11 (7-12%) |  |
| Unknown | 27 (3%) | 21-26 (4%) | ≤5 (≤6%) |  |
| Rural Status^2^ |  |  |  | <0.001 |
| No | 671 (85%) | 608 (87%) | 63 (71%) |  |
| Yes | 110-115 (15%) | 89 (13%) | 22-27 (25-30%) |  |
| Unknown | ≤5 (0%) | 0 (0%) | ≤5 (≤6%) |  |
| Charlson comorbidity score |  |  |  | 0.602 |
| 0 | 514 (65%) | 458 (66%) | 56 (63%) |  |
| 1+ | 272 (35%) | 239 (34%) | 33 (37%) |  |
| Pelvic lymph node count |  |  |  | 0.095 |
| Mean/Median | 13/11 | 13/11 | 14/13 |  |
| ≤13 | 450 (57%) | 401 (58%) | 49 (55%) |  |
| >13 | 293 (37%) | 252-257 (36%) | 37-42 (42-47%) |  |
| Not accessed | 43 (5%) | 40-45 (6%) | ≤5 (≤6%) |  |
| ***System-related*** |  |  |  |  |
| Teaching hospital |  |  |  | <0.001 |
| No | 369 (47%) | 347 (50%) | 22 (25%) |  |
| Yes | 417 (53%) | 350 (50%) | 67 (75%) |  |
| Surgeon volume, quartile^3^ |  |  |  | 0.001 |
| Q1 | 190 (24%) | 174 (25%) | 16 (18%) |  |
| Q2 | 228 (29%) | 208 (30%) | 20 (22%) |  |
| Q3 | 198 (25%) | 176 (25%) | 22 (25%) |  |
| Q4 | 167-172 (22%) | 139 (20%) | 28-33 (31-37%) |  |
| Unknown | ≤5 (0%) | 0 (0%) | ≤5 (≤6%) |  |
| Hospital volume, quartile^3^ |  |  |  | <0.001 |
| Q1 | 195 (25%) | 173 (25%) | 22 (25%) |  |
| Q2 | 212 (27%) | 212 (30%) | 0 (0%) |  |
| Q3 | 239 (30%) | 239 (34%) | 0 (0%) |  |
| Q4 | 140 (18%) | 73 (10%) | 67 (75%) |  |

Abbreviations: SES, socioeconomic status.

As per Institute of Clinical Evaluative Sciences policy, cells were suppressed to ensure that precise small cell values cannot be determined.

^1^Socioeconomic status, Quintile 1 represents communities where the poorest 20% of the Ontario population resided. SES data were not available for 27 patients.

^2^Rural status is assigned if a residence postal code is found in a community with <10 000 people. Rural status data were not available for ≤5 patients.

^3^Surgeon and hospital volume quartile 1 represent the lowest surgeon and hospital volumes. Surgeon volume data were unavailable for ≤5 patients.
